# Medical Schools

**Published:** 1881-09

**Authors:** W. R. Monroe

**Affiliations:** Baltimore


ARTICLE III.

### MEDICAL SCHOOLS.

W. R. MONROE, M. D., BALTIMORE.

The primary object of the Schools should be, not to make many Doctors, for they are too numerous already, especially in this country, but to make good doctors. They should seek to turn out competent young men, well educated in the healing art, and possessing good judgment, good manners, and good morals or a good religious training. Young men of piety, dignity and industry will succeed anywhere and in any calling, especially in that of medicine. Such men are a blessing to humanity.

And hence, the schools should not seek to become money-making institutions, but higher motives should prompt every teacher of Medicine to elevate the standard of a medical education by imparting a more thorough knowledge of its principles to his students apart from a pecuniary consideration. Any professor who lectures merely for a salary, or because it pays him liberally, ought to resign immediately and devote his time to something else. It may be true, that in any calling "The laborer is worthy of his hire," which originally referred to those who devoted their life-work to God's service, should be voluntarily supported by those to whom they ministered. But in Theology or Medicine whoever teaches or practices for money only should vacate his position and his profession. It is not hereby implied that there should be no medical fees for the teacher or practitioner, but, on the contrary, students of Medicine should pay for their medical education, and restored invalids should pay for their health-restoring agencies. But the love of God, and sympathy or love for suffering humanity, should be the chief motive for all those following the pursuit either of Medicine or Theology. The Divine promise has already been made for all the devotees among God's people, to the relief and salvation of the afflicted races of the earth: "Trust in the Lord and do good, so shalt thou dwell in the land, and verily thou shalt be fed."

A mercenary doctor or a mercenary preacher must be an abomination in the sight of God. Away with such men, who under the guise of doing good in restoring my health, and saving my soul, would fleece me out of all the means of supporting myself and my family!

> "Who will not give
> Some portion of his ease, his blood, his wealth,
> For other's good, is a poor frozen churl."

As the Medical profession is recognized as a sacred one, and as there is not much hope for reformation of old sinners in the profession, ought not the schools to seek out good young men as proper subjects to be taught the healing art? It is said the Great Physician "went about teaching and preaching the gospel of the kingdom, *and healing all manner of sickness and all manner of disease* among the people." Here is evidence of the sacredness of healing as well as that of preaching. We would not, however, advocate the combination or unity of the two professions in the same persons as a rule, but we contend that the practice of medicine is second only to that of the Ministry in its sacredness, for the health and the lives of the people are placed in the hands of physicians. Can you make good doctors out of bad men? Better make the tree good first, before you expect to grow good fruit thereon.

### Medical Classes.

A medical class should be composed of select young men. They should be selected from among good young men, well trained, educated, and of studious habits. All others making applicaticn should be sent home, or remanded back for a year's preparation in moral, mental and physical culture before matriculating in a medical college. All the students of a college should be on an equality in the eyes of every member of the Faculty. There should be manifested no preference or partiality towards any in the school. Where large numbers attend the lectures in any one college great inconvenience is experienced by many who necessarily have to sit remotely from the speaker. In large classes those seated nearest the lecturer's desk or the operating table can see and hear to greater advantage than those sitting more remote. Hence the necessity for a rule regulating the sittings so as to equalize the privileges of all the students. An exchange can be made by alternating the daily sittings, so that one-half the class shall sit near one day and then the other half occupy the same seats the next day. Or a better plan would be by an alphabetical arrangement: Let all the A's occupy the nearest seats on the first day, then next in order the B's, and so on down to the last on the alphabet, who will occupy the remotest seats on that day. On the second day B. occupies the nearest, while A. falls back to the rear. The third, C. takes the first seats, and B. the last, and so continues day by day until the last on the alphabetical list reaches the front seats; then begin again with A, and so continue in rotation during the entire session, and then all the students will be equally accomodated.

### The Time for Lectures.

It is the custom in many of the schools to have six or seven lectures daily of one hour each in length, besides the time required for demonstration in Anatomy. Here are five solid hours from 9 in the morning until two in the afternoon, add then two in the afternoon and evening, making a total of seven solid hours of lectures on seven different subjects every day. If societies have been organized for the prevention of cruelty to animals, and cruelty to children, ought not another one be organized for prevention of cruelty to medical students? Where is Mr. Bergh?

After this daily cramming process of seven long hours of lectures, what time has the student for review, for thought, for reading up and posting himself upon the various subjects discussed during the day? Or, what time has he for recreation and rest, so essential to a tired brain? People complain when compelled to listen to one sermon one hour long, on one day in the week; what cause for complaint medical students have for enduring seven hours a day for six days in the week?

No wonder they become mental dyspeptics, with seven intellectual meals every day of one hour each in duration.

Shakspeare aptly says:

> "A surfeit of the sweetest things
> The deepest loathing to the stomach brings."

Three public lectures daily ought to be quite sufficient, besides the time required for clinical instruction, and demonstration in Anatomy. Or, say, three lectures from 9 to 12 in the forenoon, with one or two hours additional during the afternoon or evening for clinics and demonstration.

### Quiz Classes an Abomination.

A bad custom has been prevailing during the past few years in some colleges, by the organization of quiz classes by certain members of the faculty. Such classes might be admissible when under the control of medical men outside of a college faculty, but a moment's reflection should convince anyone of the impropriety and unfairness of any member of the faculty conducting a quiz class. In every medical catalogue the duty of every professor is set forth, with the sum total to be paid by each student for his college education. When the students comply with the terms published, it is the incumbent duty of each and every professor to do all he can to advance the welfare and knowledge of each one in the school to the extent of his respective ability, without any extra consideration. But when a professor advertises for a quiz class at $25 or $50 each, additional, he is doing a gross injustice to all those students who have paid the regular fees of the college, by wringing out of each one who joins his class this extra fee (which sometimes may amount to a large sum; if his class reaches fifty the quiz professor gets from $1,250 to $2,500, besides his regular fees) not a dollar of which is he rightfully entitled to. And for what does he get these extra fees? Why, just for what the students are all entitled to without it. It is injustice! It is robbery!

"For when a smiling face doth cloak deceit,
It is our duty to expose the cheat."

And there is another evil growing out of Prof. Quiz's class; it is a species of bribery to insure these quiz class members success upon their final examination. Young men anxious to graduate, as all students of the second year generally are, will make almost any sacrifice or effort to bite at this bait. The money must be raised from some source, if possible, to get the influence of the vote of this professor at the final examination. These quiz class students are all posted on certain questions for the green room. And on the final day of account, all the other professors are informed that all these boys were members of Prof. Quiz's class, and they will be apt to pass them all, *nolens volens*, out of respect to their brother professor. What a farce! And, what injustice has been done to those young gentlemen who were not disposed to become members of professor Quiz's class. A distinction has been made and discrimination against the latter named gentlemen.

The entire medical school should be a quiz class, and every member of the faculty should be a quiz, but for no extra charge, as they have already been paid for all the services they should render. A good plan is, for each professor to spend the first ten or fifteen minutes of his lecture hour in brief questions on his previous lecture. This refreshens the memory of the student and fixes in his mind important facts he had heard the day before.

In the final examination of students for the degree of Doctor of Medicine, no sets of questions from Ludlow's Manual or from any other source should be selected. Those who pass out of the green room first generally post the remainder upon the questions likely to be proposed. The professor in each department should have a thousand questions at his command, and if there be one hundred candidates he will have ten original questions for each one.

"Learning by study must be won;
'Twas ne'er entailed from sire to son."

